# Evaluation of growth hormone co-treatment in *in vitro* fertilization in patients responding better to the GnRH antagonist short protocol

**DOI:** 10.5935/1518-0557.20190083

**Published:** 2020

**Authors:** José Fernando de Macedo, Maristela Rodrigues Oliveira, Luiz Mauro Oliveira Gomes, Gustavo Capinzaiki de Macedo, Giovanna Capinzaiki de Macedo, Daniela Oliveira Gomes, Olga Goiana Martins, Bruna Oliveira Ambrogi, Sandra Irene Sprogis dos Santos

**Affiliations:** 1 Reproferty (Human Reproduction), São José dos Campos, SP, Brazil; 2 Christian Life Fundation-FUNVIC, Pindamonhangaba, SP, Brazil

**Keywords:** growth hormone, *in vitro* fertilization, bad responders, assisted reproduction

## Abstract

**Objective:**

The present study aims at evaluating the results obtained after *in vitro* fertilization in bad responders, using controlled ovarian hyperstimulation together with the use of gonadotrophin releasing hormone (GnRH) antagonist (cetrorelix acetate) in a short protocol.

**Methods:**

This is an analytical, longitudinal, retrospective and controlled study involving patients who underwent *in vitro* fertilization (IVF) procedures in the assisted reproduction program of the Reproferty clinic, in the municipality of São José dos Campos/SP, from January 2012 to December 2016. We collected the data obtained from the medical records of patients considered to have undergone controlled ovarian hyperstimulation using GnRH antagonist (cetrorelix acetate) and Growth Hormone (GH) in a short cycle protocol. The patients considered controls were those submitted to the same hyperstimulation process, without using GH.

**Results:**

There were significant differences in the following analyzed parameters: gonadotrophin regimen dose, stimulation duration, and estradiol levels on the day of HCG administration, number of follicles, number of retrieved oocytes, number of mature oocytes and number of good-quality embryos. On the other hand, the GH administration was not significant in the number of cycles that achieved transfer, the number of embryos transferred and the number of frozen cycles. In the case group, there was no increase in the number of cycles that reached pregnancy rate βhCG+; however, the clinical pregnancy rates and live birth rates were significant.

**Conclusion:**

The present investigation demonstrated that GH administration as a supplement in poor responders improves the majority of the parameters to achieve a full term pregnancy in these patients.

## INTRODUCTION

Poor ovarian response has been increasingly common and frequently seen in assisted reproduction clinics. Clinical pregnancy rates remain low despite the use of different effective ovulation stimulation protocols.

There is still no clear consensus on the definition of poor responders; however, the European Society of Human Reproduction and Embryology (ESHRE) suggested the Bologna criteria in 2011, which includes at least two of the following three characteristics for such a poor ovarian response: age greater than 40 years; the number of oocytes previously recovered equal to or less than three; low ovarian reserve test scores (Ferraretti *et al*., 2011).

According to different studies, 9% to 24% of patients who underwent *in vitro* fertilization and embryo transfer (IVF-ET) were poor responders, resulting in low pregnancy rates; that is, between 2% and 4% (Lattes *et al*., 2015; Bassiouny *et al*., 2016; Aghahosseini *et al*., 2011).

Thus, there are different interventions to improve outcomes in these patients, such as the addition of growth hormone (GH) as adjuvant in pacing protocols (Surrey & Schoolcraft, 2000; Shaker *et al*., 1992; Chen *et al*., 2013).

GH is a polypeptide hormone that is described as modulating the follicle stimulating hormone (FSH) action on ovary granulocyte cells, regulating the local synthesis of Insulin-1 (IGF-1) growth factor, also involving follicular and oocyte maturation (Tesarik *et al*., 2005; Hassan *et al*., 2001; Giampietro *et al*., 2009).

On the other hand, growth hormone receptors are often expressed in the plasma membranes of granulosa cells, ovarian stromal cells and in fetal and pre-pubertal ovules (Abir *et al*., 2008). Therefore, reports such as these, suggest that GH may interact with these protein receptors, demonstrating their influence on ovarian function.

GH as an adjuvant therapy that has been in use since the 1990's, and several investigations have shown that co-treatment involving GH and gonadotrophin has a potential effect on ovulation induction (Li *et al*., 2017; Sugaya *et al*., 2003; Gerkowicz *et al*., 2015).

GH supplementation has been used in poor responder patients, including different strategies for controlled ovarian hyperstimulation (COH), with long lasting protocols, administering agonist gonadotrophin releasing hormone (GnRH) and short protocols using antagonist GnRH at doses ranging from 4 to 24 IU (Bassiouny *et al*., 2016; Dunne *et al*., 2015).

The present study aims at evaluating the results obtained after *in vitro* fertilization in bad responders, using controlled ovarian hyperstimulation with GnRH antagonist (cetrorelix acetate) in the short protocol.

The specific objectives were to compare the control group with the group that used GH supplementation in relation to: FSH dosage; treatment duration; estradiol level on the day of hCG administration; number of ovarian follicles produced above 16mm in diameter; number of oocytes recovered; number of mature oocytes; number of fertilized oocytes; number of good quality embryos; number of cycles that reached transfer; number of surplus embryos to be frozen, percentage βhCG+ and clinical pregnancy; percentage of live births.

## MATERIALS AND METHODS

This is a retrospective, longitudinal, and analytical study involving 176 patients who underwent IVF procedures in the assisted reproduction program of the Reproferty clinic, in the municipality of São José dos Campos/SP, from January 2012 to December 2016. The study was submitted to the Ethics and Research Committee, and approved under protocol number: 2.500.990.

The inclusion criteria were poor responders with advanced maternal age (≥ 40 years); poor response in a prior conventional stimulation cycle; altered ovarian reserve tests. Exclusion criteria involved those patients who did not require treatment with GH.

We collected the data from patients' charts considered as cases that underwent controlled ovarian hyperstimulation using GnRH antagonist (cetrorelix acetate) according to the short protocol and GH use. The patients considered controls were those submitted to the same hyperstimulation process, but did not react with GH.

Ovarian hyperstimulation of the studied cases was carried out with gonadotropin administration with adjusted doses according to the clinical response. We administered human chorionic gonadotrophin (hCG) in these patients to trigger ovulation (Trigger) when the follicles reached values higher than 17mm in diameter. The administered dose of GH was 4 IU daily, initiated on the second or third day of the cycle until the day of hCG administration. The following results were also evaluated: FSH dosage; treatment duration; estradiol level on the day of hCG administration; number of ovarian follicles produced bearing over 16 mm in diameter; number of oocytes recovered; number of mature oocytes; number of fertilized oocytes.

We analyzed embryo quality according to the morphological evaluation system for oocytes and embryos recommended by the Istanbul Consensus of 2011 (Alpha Scientists in Reproductive Medicine and ESHRE Special Interest Group of Embryology, 2011) and blastocysts, using the degree of morphology from Schoolcraft *et al*. (1999). Good-quality embryos were those classified as "A" and "B". We also factored in the number of cycles that reached transfer; number of surplus embryos to be frozen, number of embryos transferred, clinical and chemical pregnancy rates and live birth rate.

We plotted the data using the mean and standard deviations (X ± S) of the case and control groups. We compared the mean from the two groups using the T-test unmatched with the Welch correction. A *p* value <0.05 was statistically significant.

## RESULTS

From the 176 charts analyzed, 127 patients were treated with GH and the 49 medical records of patients who did not undergo GH treatment were used as controls.

There were significant differences in the following parameters analyzed: gonadotrophin regimen dose, stimulation duration, estradiol levels on the day of HCG administration, number of follicles, number of retrieved oocytes, number of mature oocytes and number of good quality embryos (Table 1).

**Table 1:** Characteristics of cases and controls in relation to GH administration

Variable studied (unit)	Group I (n=127)	Group II (n=49)	*P value*
FSH Dosage (UI)	2655±50.03	3214±132.70	0.0002
Stimulus duration (days)	9.60±0.12	10.55±0.17	0.0001
E2 level (pg/mL)	1243±44.42	983±76.77	0.0044
Follicles > 16 mm (*n*)	5.39±0.22	3.94±0.23	0.0001
Recovered oocytes (*n*)	3.89±0.25	2.67±0.21	0.0003
Mature oocytes (*n*)	3.03±0.22	2.33±0.19	0.0156
Fertilized oocytes (*n*)	2.42±0.19	2.08±0.19±	*p*>0.05
Embryos A+B (*n*)	1.64±0.14	1.20±0.12	0.0149
Embryos C+D (*n*)	0.74±0.11	0.90±0.14	*p*>0.05
Cycles that achieved transfer (*n*)	0.61±0.04	0.75±0.06	*p*>0.05
Transferred embryos (*n*)	1.27±0.10	1.55±0.16	*p*>0.05
Frozen embryos (*n*)	0.31±0.08	0.24±0.09	*p*>0.05

Group I – Cases;

Group II – Controls;

FSH – Follicle Stimulation Hormone;

E2 - estradiol;

On the other hand, we found that the administration of GH was not significant concerning the number of cycles that achieved transfer, the number of embryos transferred and the number of frozen cycles.

Analyzing the results described in Table 2, we noticed that in the case group, there was no increase in the number of cycles that reached Pregnancy rate βhCG+; however, the clinical pregnancy rates and live birth rates were significant.

**Table 2:** Characteristics of cases and controls according to pregnancy rates and term births.

Variable studied (unit)	Group I (n=79)	Group II (n=37)	*p value*
Pregnancy rate β hCG +	43.03±5.61	32.42±7.80	*p*>0.05
Clinical pregnancy (%)	39.24±5.53	21.62±6.86	0.049
Live births (%)	5.44±5.42	16.22±6.14	0.021

Group I – Cases;

Group II – Controls;

HG Growth Hormone

## DISCUSSION

According to the authors' conclusion associated with the Bologna consensus of 2011, after a review of several studies, there is still a lack of evidence to identify the best intervention to achieve treatment success in poor responders, due to the heterogeneity of this women’s group.

Bassiouny *et al*. (2016) evaluated the effectiveness of adding GH to the antagonist short protocol in 141 women, showing results with similar levels of significance, with the exception of the number of follicles and the quality of the embryos, parameters that the mentioned authors did not evaluate.

GH hormone supplementation was important in follicular development, stimulating the proliferation of granulosa cells. Such intervention may explain the necessity of smaller doses of FSH and the shorter duration in the days of treatment. In this sense, Hart *et al*. (2017) revealed the benefit of reducing the period of stimulation and maximizing the number of oocyte recovery. This inference is in agreement with the data obtained in the present study.

Tesarik *et al*. (2005) reported that the level of estradiol in the periovulatory follicular fluid was higher in the group that received GH as an adjuvant when compared to the placebo group, justifying that patients over 40 years of age already present a deficiency of GH due to age.

Du *et al*. (2016), using GH as a supplement in patients with normal ovulatory responses, reported an increase in estradiol on the day of HCG administration and oocyte retrieval, corroborating the data presented in Table 1, which showed statistical significance in both events.

Similar to our results, Kucuk *et al*. (2008) used GH starting in the luteal phase of the previous cycle, achieving higher estradiol serum levels on the day of HCG, in the group with supplementation. In addition, they emphasized that the GH showed an increase in the number of oocytes; however, they reported limitations in obtaining a higher rate of clinical pregnancy. The data from Tesarik *et al*. (2005) among others (Bassiouny *et al*., 2016; Hart *et al*., 2017; Eftekhar *et al*., 2013; de Ziegler *et al*., 2011) also confirmed the effect of GH on oocyte quality.

On the other hand, Eftekhar *et al*. (2013) added GH therapy to an antagonist protocol in poor responders and found no statistical significance between the groups evaluated, considering the dose of gonadotrophin used, the number of days of stimulation and the level of estradiol on the day of HCG administration.

Dunne *et al*. (2015) did not report significant differences among groups of women who underwent *in vitro* fertilization with GH administration during the luteal phase with GnRH agonist microdose protocol in normoresponders, in relation to laboratory and clinical parameters.

Data from the studies listed above contrasts with the results from the present study that suggests significantly favorable results with GH administration.

Using the same short antagonist protocol, Bassiouny *et al*. (2016) presented the same clinical parameters and did not find significant differences in the number of frozen embryos. Hart *et al*. (2017) found improvements in clinical parameters; however, there was no increase in the number of term live births with GH administration in those patients. Chen *et al*. (2013) evaluated the same topics and obtained satisfactory results in pregnancy rates. Contrasting to the studies already mentioned, other authors obtained good clinical parameters, but with no relevant values regarding the pregnancy rate (Eftekhar *et al*., 2013; Hu *et al*., 2014).

Gerkowicz *et al*. (2015) also using an antagonist protocol and administering GH at a dose of 8 IU per day, noted that the pregnancy rate was 30% *versus* 18% in the control group, reporting that more than four cycles were performed in the latter group to achieve birth when compared to the group that used GH. In this approach, Tesarik *et al*. (2005) reported a 26% increase in pregnancy rate using 12IU of GH and, Kucuk *et al*. (2008) reported rates around 32.3%.

It is noteworthy that in the present study, there was no statistical difference in βhCG+ pregnancy rate; however, a significant difference was found in the rate of clinical pregnancy and live births among those who received daily doses of 4UI GH. This effect seems to occur mainly due to the significant increase in the quality of embryos that received GH supplementation.

Lattes *et al*. (2015) showed that with the supplementation of low doses of GH, equal to 0.5 IU, there was an increase in the clinical pregnancy rate to 34.4%, which could be considered a high value compared to studies that used doses at least 16 times larger. The same authors also cited the occurrence of a significant difference in the quality of embryos, and it can be inferred that consequently more embryos were frozen. We did not used doses lower than 4 UI.

Du *et al*. (2016) used GH in patients with normal ovarian response and found an improvement in endometrium receptivity, which provides a positive impact on endometrial adhesion with consequent embryo implantation, leading to an increase in the clinical pregnancy rate. On the other hand, Duffy *et al*. (2010) found positive results only among poor responders. Among the normal responders, the use of GH did not cause the expected effect.

In our study, the rate of clinical and live birth pregnancies was significant and higher in the group that received GH as a supplement. This effect seems to occur mainly due to an increase in the development of oocytes and the action of GH in the endometrium.

Several studies whose authors (Tesarik *et al*., 2005; de Ziegler *et al*., 2011; Kolibianakis *et al*., 2009; Duffy *et al*., 2010; Mizumoto *et al*., 2017) investigated poor responders, agreed that the use of GH as an adjuvant increases the rates of live births.

## CONCLUSION

The present investigation is relevant because in those patients who received GH, there was a statistically significant difference in the parameters associated with ovulation induction potential improvement and the production of good quality embryos, resulting in a larger number of live births. However, it is important to note that further studies should be conducted to supplement information about the optimal dose, the day of the cycle to be administered and the duration of treatment to achieve an even greater number of full term births.
